# Protocol for identification of airborne asbestos fibres in the urban areas and spatio-temporal trend

**DOI:** 10.1016/j.mex.2019.09.034

**Published:** 2019-09-28

**Authors:** Yaghoub Hajizadeh, Negar Jafari, Mehdi Mokhtari, Ali Asghar Ebrahimi, Ali Abdolahnejad

**Affiliations:** a Department of Environmental Health Engineering, Faculty of Health, Environmental Research Center, Research Institute for Primordial Prevention of Non-Communicable Disease, Isfahan University of Medical Sciences, Isfahan, Iran; b Environmental Science and Technology Research Centre, Department of Environmental Health Engineering, Shahid Sadoughi University of Medical Sciences, Yazd, Iran

**Keywords:** Airborne, Asbestos fibres, Monitoring, Spatio-temporal trend

## Abstract

Asbestos is classified as a hazardous pollutants between the airborne particles that cause diseases such as lung fibrosis (asbestosis). This protocol describes an integrated method for determination of asbestos fibres concentration and its temporal-spatial trends in the air of urban areas. To do this, 60 samples were gathered from various areas of Yazd city with low, moderate and high traffic. For analysis of asbestos fibres in the samples scanning electron microscopy (SEM) and energy-dispersive X-ray (EDX) were utilized. The spatial and temporal variation of asbestos fibres concentration was carried out by ArcGIS 10 analysis. The Inverse Distance Weighting (IDW) method was used to draw asbestos fibres distribution maps. The interpolation of asbestos fibre by IDW method indicated that the distribution of the fibres in summer and winter were followed almost the similar pattern. However, the distribution of asbestos fibre concentrations in the direction of southeast to the northwest of the city was higher than that in the other areas due to high vehicular traffic.

## Graphical abstract

**Figure:**
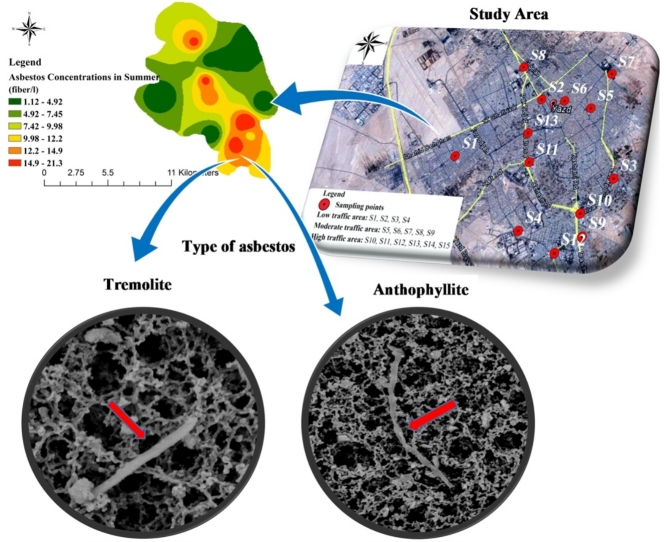

**Specifications Table**

**Table:** 

Subject Area:	*Environmental Science*
More specific subject area:	Air pollution monitoring
Protocol name:	Identification of airborne asbestos fibres in the urban areas and spatio-temporal trend
Reagents/tools:	*Scanning electron microscopy (SEM) [model WEGA/ TESCAN, Czech Republic] coupled with Energy-dispersive X-ray (EDX) system was utilized to distinct the asbestos fibres from non-asbestos ones, and it also was used to identify the types of fibres. Concentration maps were drawn using ArcGIS 10 to Show the spatial distribution.*
Experimental design:	*All sampling and analysis were conducted according to BS ISO14966 method* [1,2].
Trial registration:	*Not required*
Ethics:	*Not required*

**Value of the Protocol**

**Table:** 

• *Asbestos is a group of natural fibrous minerals that divided into two main groups of serpentine (includes chrysotile) and amphibole (includes crocidolite, anthophyllite, and amosite(* [3]. • *All types of asbestos are dangerous to human health, but the hazards of crocidolite are higher than others* [4]. • *Asbestos is a carcinogen (IARC Group 1) that Exposure to high concentration of asbestos fibres in the environment can cause many diseases such as pulmonary fibrosis which is the malignant mesothelioma and lung cancer* [[5], [6], [7], [8]]. • *There is no doubt that the inhalation of asbestos can cause human cancers. So due to the presence of various industrial resources around the city, the usage of asbestos in automobile clutches and brake pads and the inadequate information about the presence of asbestos in outdoor air of Yazd, the regular measurements of asbestos fibres in ambient air of the city, seem necessary* [4]. • *The obtained data about asbestos fibres with Arc GIS can be used for better understanding of asbestos concentration in the urban areas in association with traffic volume, population density, the industrial sources of air pollutants in the study area* [[9], [10], [11]]. • *Based on our study data, the concentration of asbestos fibres in the Yazd city is comparable with the recommended standards by WHO (based on the SEM analysis) in the ambient air* [11].

## Description of protocol

### Study area

The old and historic city of Yazd, has a population of more than 656,000 people with an area of 140 km^2^ and an altitude of 1230 m above the sea level located in the center of Iran (31° 88′ N, 54° 360′ E) [4]. This city with an average annual precipitation of 60 mm and with summer temperatures upper 40 °C is one of the warmest areas in the central desert of Iran.

### Samples collection

In this study, Yazd city according to information obtained from *Traffic and Transportation Deputy of Yazd Municipality*, the points of (S1–S4), (S5–S9) and (S10–S15) were divided into three low, moderate and high traffic areas, respectively. So, 15 sampling points were selected in these regions in the summer and winter seasons. From each sampling point 4 samples with a time interval of 45 days were gathered (60 samples) during warm and cold seasons at a height of 2.5 m from ground and near the main streets.

Air sampling was carried out by personal sampling pump (SKC Ltd MCS Flite, Swedish) with a flow rate of 10 L/min for 4–6 h, according to NIOSH Method 7400 [12]. The samples were collected on mixed cellulose ester membrane filters (type MCE; the diameter: 25 mm; the pore size: 0.8 μm; lot. No. 12557- 7DC-163) located on an SKC filter holder.

### Analytical methods

After preparation of the MCE filter, according to BS ISO14966 method, for identifying the asbestos fibres in the air samples, the scanning electron microscopy (SEM) [model WEGA/TESCAN, Czech Republic] was used [1,2]. After the fixation of the sample on holder using double-sided adhesive, samples were set in the vacuum coating apparatus (EMITECH K450X, England) for gold coating of filters with about 100 Å thick. Afterward, approximately all graticules of MCE filter surface was analyzed by SEM with the magnifications of 2000× or greater. The fibres with the following specifications: the length >5 μm, the diameter <3 μm and the length to diameter ratio of 3:1 were determined as the asbestos fibres. Then, to ensure and confirm the accuracy of the results of the study, in similar conditions, a sample was analyzed in three times by another SEM device, which was not observed significant difference among the results (p < 0.05). Energy-dispersive X-ray (EDX) system coupled with SEM was used to identify the types of fibre and distinguish asbestos fibres from non-asbestos fibres [4,13]. So, the Concentration of asbestos fibres on the MCE filter was determined using the following equation: [2]

$${\text{C}}_{\text{S}\text{E}\text{M}}=\frac{1000\;\text{N}\;\text{A}}{\text{V}\;\text{n}\;\text{a}}$$

Where, C: the concentration of asbestos fibres (fiber/ml), N: The number of fibres counted on filter, A: Effective collecting area of filter (Approximately 385 mm^2^), V: volume of air sampled (liters), n: number of fields (photos) counted on the filter and a: Calibrated surface area (mm^2^)

### Temporal and spatial analysis method

**Fig. 1:**
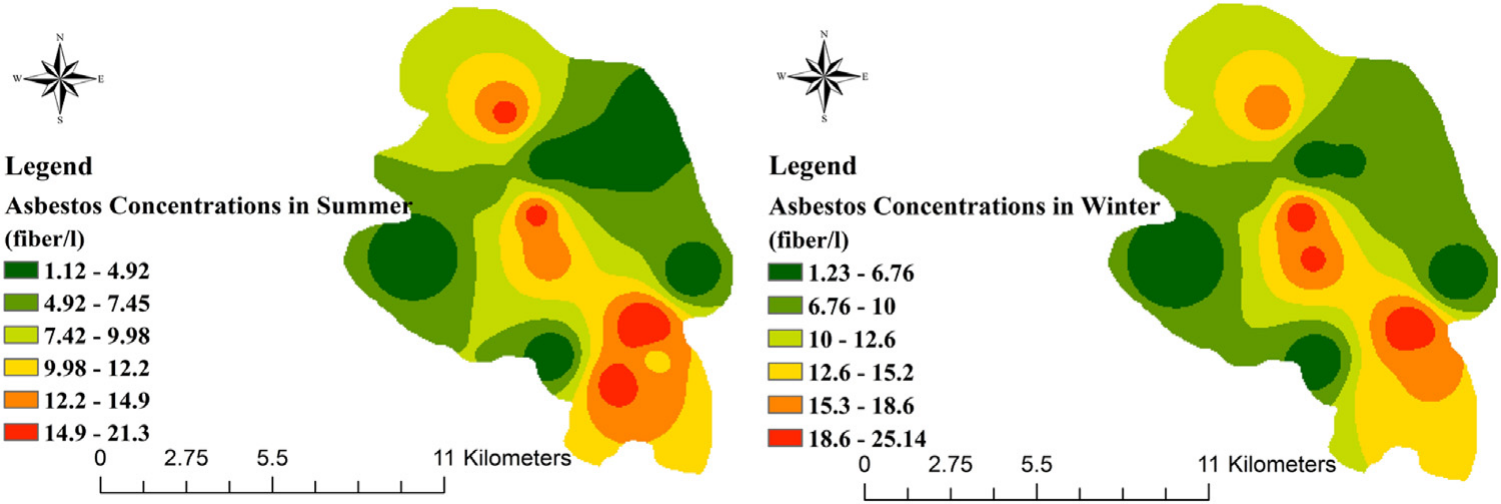
The distribution of asbestos fibres in the sampling points in summer and winter drawn by GIS.

The geometric mean of asbestos fibres concentrations in the sampling stations is presented in Table 1. The interpolation of asbestos fibre by IDW method indicated that the distribution of the fibres in summer and winter were followed almost the similar pattern. The distribution of asbestos concentrations in the direction of southeast to the northwest of the city due to high vehicular traffic was higher than that in the other areas (Fig. 1). Statistical analysis indicated that there was a significant difference among the concentration of asbestos in low and high traffic areas (p < 0.0001). The results show that heavy traffic can be one of the main source of asbestos fibres release to the urban air due to much successive braking and clutching of cars.

**Table 1:** Airborne asbestos fibre concentrations in the sampling points.

Sampling station	Area	Asbestos fibres concentration ± SD (fibre/l)
**1**	**Low traffic**	1.17 ± 2.24
**2**		2.48 ± 0.59
**3**		1.47 ± 2.79
**4**		1.62 ± 3.41
**5**	**moderate traffic**	6.42 ± 2.76
**6**		3.72 ± 2.25
**7**		5.36 ± 1.09
**8**		6.33 ± 1.23
**9**		7.08 ± 2.14
**10**	**High traffic**	17.14 ± 0.53
**11**		14.25 ± 2.92
**12**		23.22 ± 3.14
**13**		17.24 ± 1.72
**14**		15.94 ± 0.98
**15**		19.26 ± 2.56
**Mean**		9.51 ± 2.03

### Identification the type of asbestos fibres

**Fig. 2:**
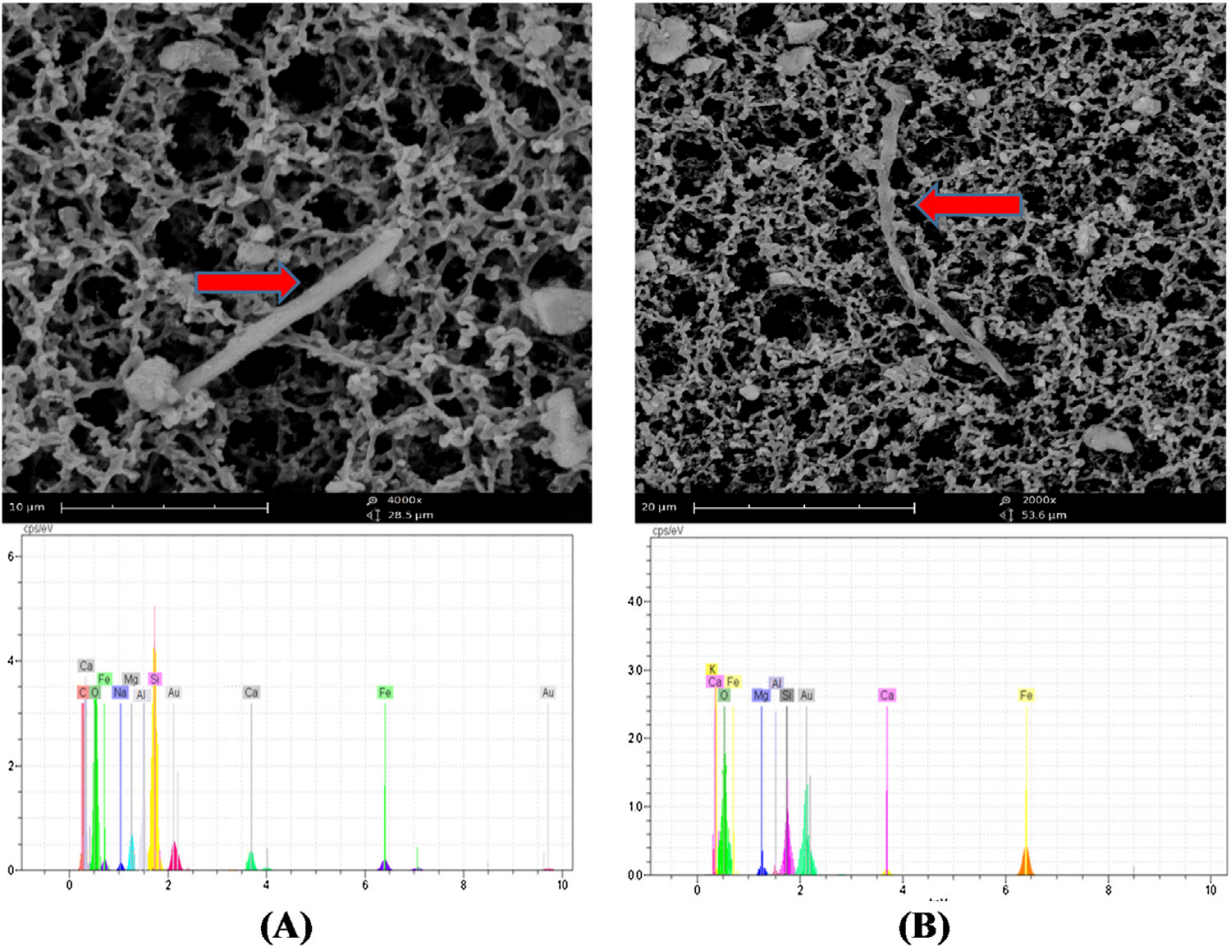
SEM image and EDX spectrum of the airborne Tremolite (A) and Anthophyllite (B) fibres.

The morphology and chemical component of asbestos fibres obtained by EDX showed that about 70% of all identified fibres were asbestos and 30% were non-asbestos fibres. For example, the specific peaks of the elements such as silica, magnesium, calcium, and iron correspond to the chemical properties of the tremolite type of asbestos fibres (Fig. 2). Also, the asbestos fibres with the distinct peaks of silica, magnesium and iron, and the Mg/Si ratio of 0.25, is recognized as anthophyllite fibres (Fig. 2).
